# Genetic Diversity of the Endemic and Medicinally Important Plant *Rheum officinale* as Revealed by Inter-Simpe Sequence Repeat (ISSR) Markers

**DOI:** 10.3390/ijms13033900

**Published:** 2012-03-22

**Authors:** Xu-Mei Wang, Xiao-Qi Hou, Yu-Qu Zhang, Rui Yang, Shi-Fang Feng, Yan Li, Yi Ren

**Affiliations:** 1 College of Medicine, Xi’an Jiaotong University, Xi’an 710061, China; E-Mails: yangruixjtu@yahoo.cn (R.Y.); Liyan3.2.1@stu.xjtu.edu.cn (Y.L.); 2 College of Life Sciences, Shaanxi Normal University, Xi’an 710062, China; E-Mails: houxiaoqi2012@stu.snnu.edu.cn (X.-Q.H.); waterfling@stu.snnu.edu.cn (Y.-Q.Z); fshfma@163.com (S.-F.F); renyi@snnu.edu.cn (Y.R.)

**Keywords:** *Rheum officinale*, inter-simple sequence repeat (ISSR), genetic diversity, genetic differentiation, conservation strategy

## Abstract

*Rheum officinale* Baill., an important but endangered medicinal herb, is endemic to China. Inter-simple sequence repeat (ISSR) markers were employed to investigate the genetic diversity and differentiation of 12 populations of *R. officinale*. Thirteen selected primers yielded 189 bright and discernible bands, with an average of 14.54 per primer. The genetic diversity was low at the population level, but pretty high at the species level (*H* = 0.1008, *I* = 0.1505, *PPB* = 28.95% *vs. H* = 0.3341, *I* = 0.5000, *PPB* = 95.24%, respectively) by POPGENE analysis. Analysis of molecular variance (AMOVA) showed that the genetic variation was found mainly among populations (74.38%), in line with the limited gene flow (*N_m_* = 0.2766) among populations. Mantel test revealed a significant correlation between genetic and geographic distances (*r* = 0.5381, *P* = 0.002), indicating the role of geographic isolation in shaping the present population genetic structure. Both Bayesian analysis and UPGMA cluster analysis demonstrated the similar results. Our results imply that the conservation efforts should aim to preserve all the extant populations of this endangered species, and cultivation is proposed in this study.

## 1. Introduction

To estimate accurately of the genetic diversity of a species is an important element in establishing conservation programs [1–3], because the ability of a species to respond adaptively to environmental changes depends on the level of genetic variability it contains [4]. Therefore, assessing the level and distribution of genetic diversity are crucial for management and the development of effective conservation strategies. Faced with the problem of preserving rare and endangered species, great concerns also should be concentrated on the endemic species with restricted geographic distribution.

*Rheum officinale* Baill. (Polygonaceae) is a perennial herb, the dried roots and rhizomes of which are called rhubarb (Da Huang in Chinese). Rhubarb is a widely used traditional Chinese medicine having many pharmacological actions, such as purgation, anti-inflammatory, antibacterial, antipyretic, and anticancer effects [5–8]. As described in the Chinese Pharmacopoeia, rhubarb also consists of the roots and rhizomes of *R. palmatum* L. and *R. tanguticum* Maxim. ex Balf. [9]. The three genuine species of rhubarb are closely related and monophyletic [10] and endemic to China [11]. As the usage of rhubarb has increased in recent decades, the wild resources have been severely destroyed. Among three genuine species of rhubarb, *R. tanguticum* has the best pharmacological effect and the most limited distribution in Qinghai, Gansu, Sichuan and Ningxia provinces. Compared with *R. tanguticum*, *R. officinale* has a weaker pharmacological effect and wider distribution in western Hubei, western Henan, southern Shaanxi, northern Sichuan, southern Chongqing, northern Guizhou, southeastern of Gansu and northwestern of Yunnan provinces. The distribution of *R. palmatum* is overlapped with the other two species [11,12]. Because of the overexploitation and the limited distribution, *R. tanguticum* has become endangered and was listed in the China higher plants endangered list [13,14]. The wild resources of *R. officinale* face great pressure exacerbated by the reduction in the individual of *R. tanguticum*. In addition to the increasing medicinal usage of *R. officinale*, the usage of *R. officinale* has recently been extended to a functional food and plant protecting agent [15]. The habitat of *R. officinale* is limited to altitudes ranging from 1100 m to 4600 m, whereby its altitudinal range does not exceed 600 m on any mountain, and it grows generally at the forest edge of the hills and rarely in the forests or in the valleys near the rivers. Because of the deterioration of the habitat of *R. officinale*, the individuals of this species are decreasing annually, thus the wild resources of it are more easily destroyed. Therefore, it can be predicted that the next original medicinal rhubarb plant which will be endangered will be *R. officinale*. To date, previous studies of *R. officinale* have mainly focused on its distribution [12,16], components analysis [17,18] and pharmacological properties [8,19,20]. Although the diversity of *R. tanguticum* had been studied by a few researchers [13,14], the genetic diversity and population structure of *R. officinale* remains unknown. In order to conserve and select excellent germplasm for the cultivation in *R. officinale*, the study on the genetic diversity for this species is becoming necessary and timely.

Of the various molecular markers, ISSR (inter-simple sequence repeat) has a few advantages because ISSR primers anneal directly to simple sequence repeat and thus, unlike SSR markers, no prior knowledge of target sequences is required for ISSR [21,22]. Also, ISSR markers, which have longer primers, allow more stringent annealing temperatures and reveal more polymorphic fragments, can be highly variable within a species and have the advantage over RAPD (random amplified polymorphic DNA) markers [23]. In addition, the cost of the analyses is relatively lower than that of some other markers such as RFLP (restricted fragment length polymorphism) and AFLP (amplified fragment length polymorphism) [24,25]. Therefore, ISSR has been widely used for population genetic studies of various plant species, including many medicinal plants [26–29].

In the present study, ISSR markers were employed to assess the genetic diversity within and among *R. officinale* populations sampled from all over its distribution. The major objectives of this study were to (1) predict the genetic diversity of the populations of *R. officinale*; (2) assess the genetic variation within and among populations; and (3) provide basic information for future conservation and management programs in the cultivation of this important medicinal species.

## 2. Results

### 2.1. Genetic Diversity

Thirteen primers were chosen to amplify a total of 199 individuals of 12 populations of *R. officinale* (Table 1). The size of all bands ranged from 200 to 2200 bp and the number of bands by each primer was from 11 to 19 with an average of 14.54 per primer (Table 2). Thirteen primers produced a total of 189 clearly identifiable bands of which 180 were polymorphic, *i.e.*, the percentage of polymorphic bands (*PPB*) for this species was 95.24% (Table 3). While at the population level, the percentage of polymorphic bands (*PPB*) ranged from 4.00% to 44.57%, with an average of 29.14%. The average effective number of alleles per locus was 1.1740. Assuming Hardy-Weinberg equilibrium, Nei’s gene diversity (*H*) varied between 0.0139 and 0.1614, with an average of 0.1014, and Shannon’s information index (*I*) ranged from 0.0213 to 0.2400, with an average of 0.1514. The values of *H* and *I* showed a similar trend to *PPB*. However, the *H* and *I* values equalled 0.3341 and 0.5000 respectively at the species level, demonstrating a relatively high level of genetic diversity (Table 3).

### 2.2. Genetic Differentiation and Relationships

The result of AMOVA showed that the percentages of genetic variation among populations were 64.38% (*G*_st_) and 74.38% (*Φ*_st_), both of which indicated that the genetic differentiation was found mainly among populations. A significant (*P* < 0.001) genetic difference was found among and within populations (Table 4). Furthermore, the level of gene flow (*N*_m_) was measured to be 0.2766 individual per generation between populations, suggesting that gene exchange between populations was very low.

**Figure 1:**
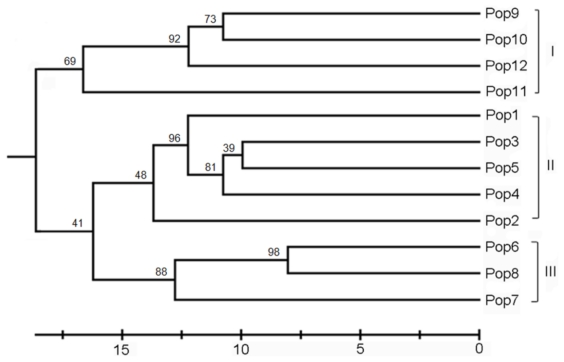
UPGMA dendrogram based on Nei’s (1978) genetic distances among populations.

**Figure 2:**
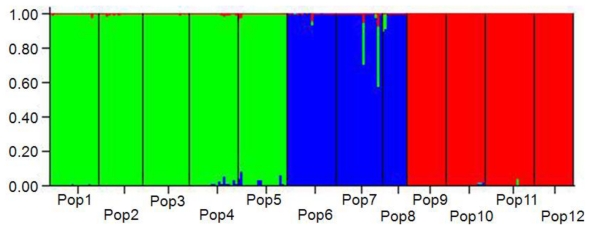
Genetic relationships among the 12 populations of *R. officinale* estimated using STRUCTURE program based on ISSR data. The model with *K* = 3 showed the highest Δ*K* value.

POPGENE analysis revealed genetic distances between populations of *R. officinale* ranged from 0.1610 (between Pop6 and Pop8) to 0.4732 (between Pop7 and Pop9) (Table 5). The UPGMA tree based on Nei’s unbiased genetic distance [30] was depicted in Figure 1, and indicated that the 12 populations were separated into three geographic groups. The populations (from Pop9 to Pop11) sampled from Yunnan province and Pop 12 from Meigu of Sichuan province formed Group I. Group II consisted of the populations from Pop1 to Pop5, and the remaining populations (from Pop6 to Pop8) formed Group III. Mantel test revealed that a significant correlation between matrices of genetic distance and of geographic distance (*r* = 0.5381, *P* = 0.002, 999 permutations). The AMOVA analysis also showed that 32.89% of total genetic variability occurred among the three geographic regions (Table 4). In the ISSR admixture analysis using STRUCTURE (Figure 2), the highest likelihood of the data was obtained when samples were clustered into three groups (*K* = 3). These groupings were entirely consistent with those of the UPGMA clustering results.

Tajima’s *D* test [31] was positive and not significant (*D* = 0.513, *P* = 0.7187). Fu’s *Fs* statistic [32] was negative and also not significant *(Fs* = −5.838, *P* = 0.0566). Thus, no clear signal of demographic expansion by Fu’s *Fs* test and Tajima’s *D* test was revealed for all the populations together. Fu’s *Fs* tests were significantly negative for the three groups (*Fs* = −6.855, *P* = 0.0112 for Group I; *Fs* = −4.575, *P* = 0.0376 for Group II and *Fs* = −1.134, *P* = 0.0037 for Group III) though Tajima’s *D* tests were positive for Group I and Group II, and negative for Group III (*P* > 0.05).

## 3. Discussion

### 3.1. Genetic Diversity

The results of the present study showed that the genetic diversity of the *R. officinale* species was *PPB* = 95.24% and *H* = 0.3341, higher than the average values of perennial herbaceous (*PPB* = 39.30%, *H* = 0.1240) and of species with endemic distribution (*PPB* = 40.00%, *H* = 0.096) [33]. The total genetic diversity of *R. officinale* was similar to that of *R. tanguticum* (*PPB* = 92.94%, *H* = 0.2689 from Hu *et al.*) [14]. The present study indicated that *R. officinale* maintained a higher genetic diversity at the species level. However, in contrast, the genetic diversity at the population level was pretty low (*PPB* = 4.00–44.57%; *H* = 0.0139–0.1654).

Although many studies have demonstrated that endangered and endemic species tend to possess low levels of genetic diversity based on ISSR data [34,35], some others have showed opposite findings [36–38]. Genetic diversity of a plant species could be affected by many factors such as distribution range, life form, breeding system and the way that its seeds disperse. A species which has had a long life, a high frequency of gene flow and many seeds tends to have high genetic diversity [33]. Species of *Rheum* is anthophilous [39]. On the other hand, the trigonous achenes of *R. officinale* have wings, so they are more easily dispersed by wind than other species of Polygonaceae. In addition, *R. officinale* is a long-living herbaceous and it can grow more than 20 years and produce large numbers of seeds every year. The germination rate of *R. officinale* is high [40]. Therefore, the long-living herbaceous habit provides more opportunity to accumulate mutant and abundant seeds and may contribute to the high amount of diversity in this species [14,41,42].

Of all the studied populations, the Pop2 population had the highest genetic diversity (*PPB* = 44.57%, *H* = 0.1614, Table 3). The Pop2 is located in Shennongjia National Nature Reserve, Hubei province. This area possesses a unique and well-preserved subtropical forest ecosystem and large numbers of rare and endangered species are on the list of UNESCO’s MAB World’s Reserve Network [43,44]. Hence, it is an ideal habitat for *R. officinale*. In contrast, genetic diversity of the Pop6 population from Mt. Jinfo in Chongqing was obviously lower than other populations. The reason might be that there are only five individuals in this population. In contrast with Shennongjia National Nature Reserve, Mt. Jinfo is a famous tourism area, and human activities frequently happen in this area. Moreover, this area is now a forest fragment surrounded by agricultural lands. Fragmentation may increase levels of inbreeding and/or genetic drift [13]. The Pop6 population is located in a smaller reserved forest compared with the other populations. According to our field investigation, the wild resources have been overexploited greatly in this site and we only found a few individuals in one cluster. From the results of genetic diversity we speculated that five individuals might be propagated from one mother plant. The results also suggested that the sampling strategy is an important factor in genetic diversity.

### 3.2. Genetic Differentiation

High genetic differentiation among populations of *R. officinale* was detected based on Nei’s gene diversity (*G*_st_ = 0.6438) and AMOVA (*Φ*_st_ = 0.7438), which is similar to the higher genetic differentiation in endangered and endemic species in *Megacodon stylophorus* (Gentianaceae) [45], *Rhodiola alsia* (Crassulaceae) [46] and *Torreya jackii* [38]. Correspondingly, the among-population differentiation (*Φ*_st_) is 0.807, 0.703 and 0.670, respectively. However, the present study showed an opposite finding to the previous studies of *R. tanguticum* [13,14]. Hu *et al.* [14] reported that the among-population differentiation coefficients were 0.3585 (*G*_st_) and 0.290 (*Φ*_st_), and that from Chen *et al.* were lower (*G*_st_ = 0.249, *Φ*_st_ = 0.2118) [13]. The reason for the contradiction between these different studies might be that the populations of *R. tanguticum* were collected only from Qinghai province in their studies. In fact, *R. tanguticum* was also distributed in Gansu, Sichuan and Ningxia provinces [12]. The sampled populations of *R. officinale* in the present study covered the entire distribution. As Pfeifer and Jetschke [47] reported geographic isolation is one major factor influencing genetic differentiation by limiting the amount of gene flow via both pollen and seeds. In the present study, the largest and mean inter-population geographic distances were 1174.91 km (Pop1 *vs.* Pop11) and 591.854 km, respectively. *R. officinale* is outcrossing and its seeds are dispersed by wind. Therefore, large geographic barriers, such as Mt. Qinling and Mt. Daba and the human activities between populations greatly hindered gene flow via seed and pollen dispersal among populations. Typically, when *N*_m_ < 1, differential selection might be very strong, and population differentiation might be maintained [48]. In *R. officinale*, *N*_m_ estimated from *G*_st_ was only 0.2766 and that is far below 1, indicating the gene flow among *R. officinale* populations with discontinuous distributions was limited and might enhance the genetic differentiation among populations. Moreover, the Mantel test revealed a significant correlation between genetic and geographic distances (*r* = 0.5381, *P* = 0.002), displaying a clear isolation of populations by geographic distances in shaping the present genetic structure of *R. officinale.* This genetic structure was further confirmed by the topology of UPGMA (Figure 1) and the Bayesian analyses (Figure 2). On the other hand, the roots and rhizomes of *R. officinale* have been overexploited in the past decades, and its habitat gradually limited to small isolated areas. For example, the size of some populations like Pop6 and Pop8 in this study is small, and only a few individuals can be found in the field. The small populations are likely to have been subjected to strong genetic drift. Genetic drift changes the distribution of genetic variation in two ways, by reducing variation within populations and by increasing differentiation between populations [49]. Meanwhile, the theory of population genetics indicates that genetic differentiation should increase in progressively smaller and more isolated populations [49,50].

In the UPGMA tree, populations were mainly divided into three geographic groups (Figure 1). Group I included the populations Pop9, Pop10, Pop11 and Pop12, while the remaining populations were clustered into two other groups. This division reflects geographic distribution pattern of these populations. The populations Pop9, Pop10, Pop11 and Pop12 are geographically distant from the other populations and are located in Hengduan mountain system (southwestern China). Group II included the populations from Pop1 to Pop5 which were sampled from central and eastern part of Mt. Qinling and eastern Mt. Daba, while Pop6, Pop7 and Pop8 sampled from Mt. Dalou (Pop 7 and Pop 8) and Pop6 from western Mt. Daba formed Group III. Populations from Mt. Hengduan which is located in the southeastern part of the Qinghai-Tibet Plateau is in the southwestern part of the distribution of *R. officinale*, whereas populations from central and eastern Mt. Qinling and eastern Mt. Daba are in the eastern part of the distribution of *R. officinale*. This finding was consistent with the results of the Bayesian analyses, which was clearly identified the same three main group clusters. Hence, the present genetic structure could be partly explained by the isolation-by-distance model [51], as indicated by the result of the Mantel test. However, the genetic differentiation among the three groups was lower than within groups. The reason of this may be that the weak expansion existed within each of the three groups respectively, as indicated by the neutrality deviation in Fu’s *Fs* tests.

Combined with the geographic distribution pattern of *R. officinale*, the genetic diversity of populations which were collected from southwestern part of its distribution (*i.e.*, Pop9-Pop12 in Mt. Hengduan) was lower than that of the populations from the central (e.g., in Mt. Dalou and western Mt. Daba) and northeastern of its distribution (central and eastern Mt. Qinling and eastern Mt. Daba). It is well known that China’s topography is divided into three terraces from the west to the east: Mt. Hengduan lies in the first terrace which mainly includes the Qinghai-Tibet Plateau, with the altitude higher than 3000 m; the second terrace refers to the eastern and northern areas out of the Qinghai-Tibet Plateau, with an average altitude of lower than 3000 m; the third terrace is mainly hills and plains in the east, with an average altitude of lower than 500 m. *R. officinale* is distributed in the first and the second terrace. Populations Pop9 to Pop12 are located in Mt. Hengduan which belongs to the first terrace, and the altitudes of those populations are higher than 3400 m. The other populations are located in the central and the northeastern area of the species’ distribution in the second terrace, with an altitude of lower than 2900 m. Compared with the second terrace, the first terrace is the youngest orogeny of the mountains in Neozoic era, and the genetic diversity of the populations located in this area is low. The reason may be that accompanying with the uplifts of Mt. Hengduan, the distribution of the species extends to the higher altitude, which might cause the disjunctive distribution of *R. officinale*. This disjunctive distribution pattern of *R. officinale* may be another reason for the present genetic structure. These areas with lower genetic diversity are not the suitable habitat for the growth of the species.

### 3.3. Implications for Conservation and Cultivation

The primary objective in nature conservation is to preserve as much as possible of the evolutionary potential of species through maintaining as much genetic diversity as possible [34]. The maintenance of genetic variation is a major objective within conservation plans for endangered species [52,53]. Information obtained in the present study provides significant implications for conservation strategies of *R. officinale*. The results reported here revealed low genetic diversity at population level and high genetic diversity at species level in *R. officinale*. High genetic differentiation that occurred among populations and might be due to limited gene flow and genetic drift. In general, this species is not genetically depauperate. The main factor responsible for this threatened species may be recent over-collection of the medicinal organs from the field, rather than a lack of overall genetic diversity. Therefore, the exploitation of wild resources should be forbidden. Considering the high genetic differentiation of *R. officinale*, preservation of any population would be insufficient to conserve all the variation in the species. Thus, the priority must be to protect all the existing populations *in situ*, especially those extant populations with high levels of genetic variation of different regions such as Pop1, Pop2 and Pop5, we suggest that their habitats be protected and the exploitation of wild resources be prevented. If *ex situ* conservation is required, samples should be collected from as many populations as possible from the whole distribution, and populations with small sizes (e.g., Pop6 and Pop8) should receive more attention. On the other hand, to meet the bulk commercial demand for this traditional medicinal plant, cultivation facilities can be established as an alternative source of raw materials. Therefore, the high genetic diversity of the materials (e.g., Pop1, Pop2 and Pop5) should be used in cultivation in order to reduce the inbreeding depression.

## 4. Experimental Section

### 4.1. Plant Sampling

Twelve populations were sampled throughout the distribution of *R. officinale*, including Henan, Hubei, Shaanxi, Sichuan, Chongqing, Guizhou and Yunnan provinces, China. 16–20 (only five and ten individuals in Chongqing and Guizhou populations, respectively) fresh leaves were collected randomly in each population, depending on accessibility and population size. Each population was positioned by a GPS and the detailed locations of the studied populations are listed in Table 1. The young leaves were stored and dried in ziplock bags with silica gel and transported back to our laboratory for DNA extraction. The voucher specimens were deposited in the Shaanxi Normal University Herbarium (SANU).

### 4.2. DNA Extraction and PCR Amplification

Total DNA was extracted from the silica gel-dried leaves using the modified 2× CTAB procedure [54]. The quality and quantity of DNA were performed by UV-spectrophotometer (ND-2000, NanoDrop, USA). DNA concentration and purity were also determined by electrophoresis on 1.0% agarose gels based on the intensities of band when compared with l kb plus DNA ladder as marker. The DNA samples were diluted to the concentration of 50 ng/μL and stored at −20 °C for use.

One hundred ISSR primers synthesized by Shanghai Sangon Biological Engineering Technology & Service (China), according to the primer set published by University of British Columbia, Canada (UBC set No.9) were used for amplification to standardize the PCR conditions. Thirteen of 100 ISSR primers produced clear, reproducible and relatively high polymorphism bands were selected for all samples of *R. officinale* (Table 2). The effects of Mg^2+^, dNTPs, DNA templates, primers concentrations and annealing temperature on the amplification were tested, and the final amplification was carried out according to our previous study for the optimized ISSR-PCR reactions of *R. officinale* [55]. PCR products were electrophoresed on 1.6% (*w*/*v*) agarose gels, in 1× TBE Buffer at 110 V for 1.5 h and stained with ethidium bromide (0.5 μg/mL). Gels with amplification fragments were visualized and photographed in UV light by using Bio-Rad Gel Documentation System (Bio-Rad Laboratories, UK). DL2000 ladder (TaKaRa Biotechnology, China) was used as DNA molecular weight.

### 4.3. Data Analysis

The amplified fragments, with the same mobility according to their molecular weight (bp), were scored in terms of a binary code as present (1) or absent (0). Only those consistently reproducible bands were scored, and smeared and weak bands were excluded. Genetic parameters including the percentage of polymorphic bands (*PPB*), observed number of alleles (*N*a), the effective number of alleles (*N*e), Nei’s gene diversity (*H*) [56], Shannon’s index (*I*) [57], Nei’s genetic identity and genetic distance [30], Nei’s genetic differentiation index among populations (*G*_ST_) [56] and gene flow (*N*m) were calculated using the computer program POPGENE version 1.32 [58]. An estimate of *Nm* among populations was computed using the formula of *N*m =0.5(1 − *G*_ST_)/*G*_ST_ [59]. The obtained genetic distance matrix was then used to perform the cluster analysis and construct the unweighted pair-group method with arithmetic average (UPGMA) dendrogram using MEGA 4.0 [60]. In addition, an analysis of molecular variance (AMOVA) procedure was used to estimate the partitioning of genetic variance among and within populations. Input data files for the AMOVA 1.55 [61] were generated using DCFA version 1.1 [62]. The significance of variance components were tested statistically by nonparametric randomization tests using 1000 permutations. Geographic distances were interpreted by the latitudes and longitudes with Mapinfo 8.0 Program. The Mantel test of genetic and geographic distances was carried out to evaluate the correlation between the two data matrices using TFPGA software [63] (computing 999 permutations). Tajima’s *D* test [31] and Fu’s *Fs* test [32] were conducted to test the neutral mutation hypothesis using ARLEQUIN Version 3.0 [64]. The demographic history of a population could be inferred by comparing such neutrality tests, given that a range expansion is suggested when Tajima’s *D* and Fu’s *Fs* are significantly negative [32].

A Bayesian analysis of ISSR population structure was performed on the entire data set using the program STRUCTURE (version 2.3) [65] to detect population structure and estimate the number of populations (*K*) in a sample and to assign individuals to one or more of these populations (*K*). The number of genetically distinct clusters (*K*) was set to vary from 1 to 12 (the total number of populations). The model was run for eight independent simulations for each *K*, used a burn-in length of 50,000 and a run length of 100,000 iterations. Following the program’s dominant marker settings, the “no admixture” model was used, and uncorrelated allele frequencies among populations were assumed. The most likely number of clusters was estimated according to the model value (Δ*K*) based on the second order rate of change, with respect to *K*, of the likelihood function, following the procedure described by Evanno *et al.* [66].

## 5. Conclusions

In summary, our results indicated that the genetic diversity of *R. officinale* was high at the species level, while the genetic diversity was low at the population level. High genetic differentiation was found mainly among populations, which may be attributed to geographic isolation by the limited gene flow and genetic drift. Cluster analysis using the UPGMA method grouped all populations into three geographic groups. Based on these findings, strategies are proposed for the conservation and cultivation of the species.

## Figures and Tables

**Table 1:** Sampling details of *R. officinale* populations in the present study.

Population	Locality	Longitude (E)	Latitude (N)	Altitude (m)	Sample size	Voucher
**Pop1**	Baotianman Reserve, Mt. Funiu, Neixiang county, Henan	33°29.179′	111°54.96′	1100	20	Xu-mei Wang 08082417
**Pop2**	Muyu, Mt. Shennongjia, Xingshan county, Hubei	31°27.088′	110°16.172′	2908	18	Xu-mei Wang and Xiao-qi Hou 09071101
**Pop3**	Doumugong, Mt. Taibai, Mei county, Shannxi	34°02.286′	107°42.869′	2841	19	Xiao-qi Hou 10072001
**Pop4**	Taibaimiao, Ningshan county, Shaanxi	33°25.406′	108°31.833′	1878	20	Xiao-qi Hou 10071301
**Pop5**	Mt. Hualong, Pingli county, Shaanxi	32°01.393′	109°21.499′	2919	20	Xiao-qi Hou 2010072801
**Pop6**	Daping, Mt. Jinfo, Nanchuan county, Chongqing	28°58.414′	107°11.023′	1412	5	Xu-mei Wang and Xiao-qi Hou 09072711
**Pop7**	Nanjiang county, Sichuan	32°35.668′	107°06.78′	1809	19	Yu-qu Zhang 10080801
**Pop8**	Hailongtun, Gaoping, Zunyi county, Guizhou	27°48.766′	106°49.097′	1252	10	Xu-mei Wang and Xiao-qi Hou 20090731
**Pop9**	Gelachang, Haba, Sanba, Xianggelila county, Yunan	27°23.219′	100°02.754′	3995	16	Xiao-qi Hou 09080718
**Pop10**	Parch house, Haba, Sanba, Xianggelila county, Yunan	27°23.741′	100°02.257′	3727	16	Xiao-qi Hou 09080720
**Pop11**	Xiaozhongdian, Xianggelila county, Yunan	27°35.118′	99°50.835′	3441	20	Xiao-qi Hou 1010202
**Pop12**	Hongxi, Meigu county, Sichuan	28°40.243′	102°58.341′	3623	16	Yu-qu Zhang and Xiao-qi Hou 09091824

**Table 2:** Inter-simple sequence repeat (ISSR) primers used for ISSR analysis in the present study, Y = (C, T); B = (C, G, T); D = (A, G, T); H = (A, G, T); V = (A, C, G).

Primer code	Sequence (5′→3′)	Annealing temperature (°C)	No. of amplified bands	No. of polymorphic bands
UBC807	(AG)_8_T	51	12	12
UBC811	(GA)_8_C	53	13	11
UBC816	(CA)_8_T	52	16	15
UBC825	(AC)_8_T	52	12	11
UBC834	(AG)_8_YT	52	14	14
UBC835	(AG)_8_YC	52	10	9
UBC836	(AG)_8_YA	52	16	16
UBC841	(GA)_8_YC	52	15	14
UBC842	(GA)_8_YG	56	11	11
UBC888	BDB(CA)_7_	52	19	18
UBC889	DBD(AC)_7_	52	17	17
UBC890	VHV(GT)_7_	56	18	17
UBC891	HVH(TG)_7_	52	16	15
**Total**	-	-	**189**	**180**

**Table 3:** Genetic diversity within populations of *R. officinale*, *N*_a_: observed number of alleles; *N*_e_: effective number of alleles; *H*: Nei’s (1973) gene diversity; *I*: Shannon’s information index; *PPB*: percentage of polymorphic bands.

Populations	*N*_a_	*N*_e_	*H*	*I*	*PPB* (%)
**Pop1**	1.4457	1.2626	0.1555	0.2332	44.57
**Pop2**	1.4457	1.2771	0.1614	0.2400	44.57
**Pop3**	1.3943	1.2210	0.1305	0.1970	39.43
**Pop4**	1.3657	1.2156	0.1251	0.1877	36.57
**Pop5**	1.4343	1.2730	0.1563	0.2318	43.43
**Pop6**	1.0400	1.0213	0.0139	0.0213	4.00
**Pop7**	1.3200	1.1129	0.0699	0.1115	32.00
**Pop8**	1.1143	1.0766	0.0435	0.0640	11.43
**Pop9**	1.2457	1.1737	0.0990	0.1447	24.57
**Pop10**	1.2229	1.1614	0.0914	0.1329	22.29
**Pop11**	1.1771	1.1190	0.0688	0.1014	17.71
**Pop12**	1.2686	1.1613	0.0940	0.1402	26.86
**Average**	1.2895	1.1730	0.1008	0.1505	28.95
**Species level**	1.9543	1.5696	0.3341	0.5000	95.24

**Table 4:** Analysis of molecular variance (AMOVA) from 12 populations of *R. officinale* using 13 inter-simple sequence repeat markers, d.f., degree of freedom; SSD, sum of squares; MSD, mean squared deviations; VC, variance component; TVP, total variance percentage;

Source of variation	d.f.	SSD	MSD	VC	TVP (%)	*P*-value *
Among populations	11	2076.62	207.66	24.37	74.38	<0.001
Within populations	187	663.00	8.39	8.39	25.62	<0.001
Among geographic regions	2	712.42	356.21	11.42	32.89%	<0.001
Within geographic regions	196	2027.20	23.30	23.30	67.11%	<0.001

* Significance tests after 1000 permutations.

**Table 5:** Nei’s (1978) unbiased measures of genetic distance (below diagonal) and genetic identity (above diagonal) between *R. officinale* populations.

Population	Pop1	Pop2	Pop3	Pop4	Pop5	Pop6	Pop7	Pop8	Pop9	Pop10	Pop11	Pop12
**Pop1**	****	0.7674	0.8084	0.7491	0.7928	0.6960	0.7165	0.7238	0.6944	0.7279	0.7072	0.7026
**Pop2**	0.2648	****	0.7455	0.7783	0.7513	0.6690	0.6904	0.6920	0.6890	0.7054	0.6778	0.6903
**Pop3**	0.2127	0.2937	****	0.8022	0.8196	0.6954	0.7258	0.7520	0.7027	0.6922	0.6784	0.7258
**Pop4**	0.2889	0.2507	0.2205	****	0.8108	0.7420	0.7408	0.7770	0.6770	0.7140	0.7289	0.7044
**Pop5**	0.2322	0.2859	0.1990	0.2098	****	0.7347	0.7313	0.7647	0.7084	0.7173	0.7350	0.6925
**Pop6**	0.3625	0.4019	0.3633	0.2984	0.3083	****	0.7499	0.8513	0.6349	0.6627	0.6383	0.6522
**Pop7**	0.3334	0.3704	0.3204	0.3000	0.3129	0.2878	****	0.7997	0.6230	0.6961	0.6543	0.6810
**Pop8**	0.3232	0.3682	0.2850	0.2523	0.2683	0.1610	0.2235	****	0.6851	0.7119	0.6642	0.6888
**Pop9**	0.3647	0.3725	0.3528	0.3900	0.3447	0.4542	0.4732	0.3783	****	0.8064	0.7108	0.7865
**Pop10**	0.3175	0.3490	0.3678	0.3369	0.3323	0.4114	0.3623	0.3398	0.2152	****	0.7364	0.7803
**Pop11**	0.3464	0.3890	0.3880	0.3162	0.3079	0.4490	0.4241	0.4092	0.3413	0.3059	****	0.7040
**Pop12**	0.3529	0.3707	0.3204	0.3504	0.3674	0.4275	0.3842	0.3728	0.2401	0.2481	0.3510	****
